# FAMILY PERCEPTIONS ABOUT ENGAGING IN PALLIATIVE CARE CONFERENCES FOR THEIR LOVED ONES WHO LIVE IN LONG-TERM CARE

**DOI:** 10.1093/geroni/igae098.2388

**Published:** 2024-12-31

**Authors:** Sharon Kaasalainen, Genevieve Thompson, Abigail Wickson-Griffiths, Paulette Hunter, Lynn McCleary, Tamara Sussman

**Affiliations:** McMaster University, Hamilton, Ontario, Canada; University of Manitoba, Winnipeg, Manitoba, Canada; University of Regina, Regina, Saskatchewan, Canada; University of Saskatchewan, Saskatoon, Saskatchewan, Canada; Brock University, St. Catharines, Ontario, Canada; McGill University, Montreal, Quebec, Canada

## Abstract

Despite the high mortality rates in long term care (LTC), most LTC homes do not have a formalized palliative program. Communication with family members or care partners is critical to prepare them for end-of-life and promotes shared decision-making. The aim of this study was to explore family perceptions about engaging in Palliative Care Conferences (PCCs) which were held for their loved one who was dying in LTC. This study was conducted in four provinces in Canada (Ontario, Manitoba, Saskatchewan, Alberta) and utilized a qualitative description design. Of the 36 family members who were interviewed, 12 were bereaved and 24 were non-bereaved. Overall, they felt that PCCs provided a venue for learning and appreciated the interdisciplinary approach. It gave them some time and space to develop stronger, more caring relationships with staff that sometimes was difficult to do during normal day-to-day activities. Family stated the PCCs were informative and promoted quality communication. They also described how the timing of PCCs and other discussions is important so they don’t feel rushed and can ‘go at their own pace’, allowing them to receive the information that they need when they are ready for it. PCCs appear to feasible and support a family-centered approach to care, which relies on strong communication. Future work needs to include a more rigorous evaluation that builds PCCs into everyday practice.

